# Ketone monoester ingestion and cognitive and physical performance during exercise-heat stress

**DOI:** 10.1080/15502783.2026.2695136

**Published:** 2026-07-16

**Authors:** Blaine S. Lints, Riccardo F. Romersi, Jenica N. Earl, Maria G. Lombardi, Katelynn T. Persaud, Noah K. Nakagawa, Chimaobim E. Martin-Diala, Gianna F. Mastrofini, Adam T. Harrison, Susan W. Yeargin, R. Davis Moore, Jose Antonio, Shawn M. Arent

**Affiliations:** a Department of Exercise Science, Arnold School of Public Health, University of South Carolina, Columbia, SC, USA; b Nova Southeastern University, Department of Health and Human Performance, Davie, FL, USA

**Keywords:** Sports nutrition, ketone monoester, dietary supplements, stimulants, cognition, tactical

## Abstract

**Background:**

Sustaining cognitive and physical performance under extreme environmental conditions is critical for special operation forces (SOF). Hyperthermia negatively impacts cognitive function due to reductions in cerebral blood flow, substrate availability, thermal tolerance, and increased cardiovascular strain. While ketone monoester have demonstrated beneficial effects on cognition, their efficacy during exercise-heat stress remains unexplored.

**Methods:**

Seventeen endurance-trained males (age = 23.8 ± 5.2 y; VO_2_max = 58.6 ± 3.2 ml·kg^−1^·min^−1^) completed a randomized, double-blind, counterbalanced, crossover study. Participants ingested 4 mg·kg^−1^ caffeine combined with either a ketone monoester (KET; (R)-3-hydroxybutyl-(R)-3-hydroxybutyrate) or an energy-matched carbohydrate control (CHO; Cluster Dextrin™). Each experimental visit consisted of 90 minutes of loaded treadmill exercise in the heat (34 °C, 45% RH) followed by a high-intensity time-to-exhaustion (TTE) test. Cognitive performance (1-back, 2-back, Dynavision reaction time [RT], object hit and avoid [OHA]), blood metabolites, average heart rate, and core body temperature (CBT) were assessed before, during, and after exercise and analyzed using linear mixed-model ANCOVA. TTE duration and maximal heart rate during TTE were analyzed using paired-samples t tests.

**Results:**

For cognitive performance, KET demonstrated significantly greater 1-back total accuracy (+2.8%, *p* = 0.001) and target accuracy (8.9%, *p* < 0.001) relative to CHO across all timepoints, Additionally, KET increased overall target discrimination (d′: + 0.3, *p* = 0.001) irrespective of task condition and timepoint. For 2-back, no overall between-condition differences were observed in total or target accuracy across time points, although task difficulty remained higher than 1-back performance overall. No between-condition differences were observed for RT or OHA outcomes. For blood metabolites, KET significantly increased circulating BHB concentrations relative to CHO at mid- and post-exercise (+1.7 and + 3.5 mmol·L^−1^, respectively; both *p* < 0.001) and reduced blood glucose by 14.7 and 27.6 mg·dL^−1^ at the same time points (*p* = 0.002 and *p* < 0.001, respectively). For average heart rate, there was a main effect of time, with heart rate lower during the first 45-minutes exercise bout than the second (−7.7 beats·min^−1^, *p* = 0.01), but no effect of condition (*p* = 0.453). CBT increased over time in both conditions (*p* < 0.001) but did not differ between KET and CHO (*p* = 0.065). TTE duration was significantly longer following KET than CHO (8.9 ± 4.6 vs 7.0 ± 2.1 min; *p* = 0.04), whereas maximal heart rate during TTE did not differ between conditions (*p* = 0.112).

**Conclusions:**

Ketone monoester ingestion combined with caffeine improved select aspects of working memory and resulted in a significantly longer time-to-exhaustion compared with carbohydrate control following prolonged, loaded exercise in the heat. However, these effects were not universal across all cognitive outcomes, as no between-condition differences were observed for 2-back, RT, or OHA. Notably, despite performance differences, average heart rate, maximal heart rate during TTE, and core temperature did not differ. Together, these findings suggest that ketone monoester and caffeine co-ingestion may support specific cognitive and physical aspects of performance during sustained exercise-heat stress without altering cardiovascular or thermal strain.

## 1. Introduction

United States (US) special operations forces (SOF) engaged in combat operations are required to sustain elevated levels of human performance in extreme environmental conditions. For example, the US military held a presence in Iraq for over 30 years, a country which regularly records temperatures exceeding 48 °C (120 °F.) Furthermore, the US military continues to maintain a presence throughout many other geographic regions with high degrees of heat strain such as Africa, South America, and Asia. Hyperthermia negatively affects cognitive performance due, in part, to decreased thermal comfort, increased cardiovascular strain, impaired metabolism, and a reduction in cerebral blood flow with subsequent substrate delivery [1–3]. Additionally, these cognitive insults are exacerbated by sustained military operations [4]. Considering the need for SOF personnel to respond rapidly and appropriately to unexpected threats, the hot conditions and subsequently altered cognitive processing routinely experienced during combat operations could have potentially catastrophic consequences at the individual, team, and operational levels.

Exercise-heat stress presents the cardiovascular system with the challenge of maintaining arterial pressure while redistributing blood volume to cutaneous tissues for cooling, as well as metabolically active tissues [5]. As thermal strain increases, the cardiovascular system is unable to adequately supply working muscles and cutaneous tissues with sufficient blood volume while continuing to maintain cerebral blood flow, cooling, and substrate delivery [6]. These physiologic alterations can result in confusion and altered states of consciousness as well as acute reductions in short-term memory and information processing [7]. Moreover, glucose, the primary energy substrate for the brain, may become less available due to impaired delivery or altered uptake during heat stress [8]. Notably, an 18% global decrease in cerebral blood flow was observed during exercise-heat stress when compared to similar exercise in normothermic conditions [9]. In many cases, these reductions are accompanied by acute decreases in short-term memory and information processing which precede delays in reaction time and impaired attention, all of which are skills paramount for SOF personnel [7].

Ketone bodies are produced endogenously by the liver in situations of insufficient glucose availability. Ketones provide an alternative substrate for cerebral metabolism, increase cerebral blood flow, and have been applied clinically to treat various neurological disorders [10] and brain injuries [11]. Further, ingestion of exogenous ketone supplements, including ketone monoester (KET), can shift an individual into a ketotic state without necessitating any form of energy restriction. Indeed, 25 g of orally ingested KET has been demonstrated to be more than sufficient to induce ketosis [12]. In a randomized controlled human trial, a ketone infusion increased plasma beta-hydroxybutyrate (BHB) concentrations to an average of 5.5 mmol/L, which was temporally related to a 30% increase in blood flow in cortical gray, occipital, temporal, frontal, and parietal brain regions [13]. Another study found that increasing blood BHB concentrations to 2.16 mmol/L was accompanied by a 39% increase in cerebral blood flow [14].

Several studies have also examined the effects of KET on cognitive performance in humans. For example, Evans and Egan (2018) noted that the decline in executive function following exhaustive exercise was attenuated by the ingestion of ketone esters when compared to a carbohydrate control [15]. In two crossover studies, researchers examined whether KET could mitigate cognitive impairment during acute hypoxia by providing an alternative cerebral substrate and supporting oxygen saturation. In a pilot trial, Coleman et al. found that KET attenuated hypoxia-induced declines in code substitution accuracy and blink rate, suggesting a neuroprotective effect [16]. Building on these findings, a subsequent trial involving military personnel demonstrated that acute KET ingestion (650 mg/kg) prior to normoxic and hypoxic exposures elevated blood BHB concentrations by >4 mM and reduced blood glucose by ~20 mg/dL. During simulated high-altitude exposure (6096 m), KET significantly attenuated declines in oxygen saturation and improved cognitive efficiency on a code substitution task by 6.8 correct responses per minute [17]. These results support the potential of KET to support cognitive resilience and physiological stability under oxygen-restricted conditions.

Lastly, military service members routinely ingest caffeine [18]. Caffeine is the most commonly used supplement in the world and has GRAS status. It has been shown to be safe in doses as high as 9 mg/kg while concurrently providing an ergogenic effect during exercise [19] and athletic endeavors [20]. More specifically, caffeine has also been shown to enhance performance during operationally relevant tasks [21], and this effect extends to multi-day, sustained operations in SOF personnel [22]. The essential role that caffeine plays in military culture, its ergogenic effects on cognitive and physical performance, and the paucity of data examining the combination of BHB and caffeine, highlights the importance of its addition to this intervention. Therefore, the purpose of this study was to determine whether ingestion of KET with caffeine would enhance cognitive and/or physical performance during exercise-heat stress compared to carbohydrate with caffeine.

## 2. Methods

A randomized, double-blind, placebo-controlled, counterbalanced, crossover design was used to compare the effects of a 4 mg/kg body mass (BM) caffeine and KET or a carbohydrate control (CHO) on cognitive performance before, during, and after fatiguing exercise in a heat chamber. Participation in the clinical trial required each participant to complete one familiarization visit and two experimental visits separated by 7–14 days. Between visits, participants were asked to continue their normal exercise regimen. For the experimental visits, the supplementation order was randomized for each participant with counterbalancing. All protocols and procedures were approved by the University of South Carolina Institutional Review Board (IRB Number: Pro00139698; approved 1/29/2025) prior to data collection. This study was registered on ClinicalTrials.gov (NCT07010666).

### 2.1. Participants

Seventeen healthy, endurance-trained males between the ages of 18–35 years old completed this clinical trial (*n* = 17; age = 23.8 ± 5.2 y; height = 177.7 ± 5.7 cm; BM = 80.1 ± 4.2 kg). All participants regularly participated in resistance and/or endurance training (≥4 days/week and ≥ 150 min/week) for at least six months prior to enrollment, and all participants met the aerobic fitness criterion for heat stress set by McLellan (VO_2max_ greater than 55 ml/kg/minutes VO_2max_ = 58.6 ± 3.2 ml/kg/min) [23]. Participants were excluded if they 1) were currently taking any stimulant medication, 2) had any known metabolic disorder (e.g. electrolyte abnormalities, diabetes, thyroid disease), 3) had a history of hepatorenal, musculoskeletal, autoimmune, or neurological disease, 4) experienced migraines, 5) had a history of heat stroke, or 6) had a known caffeine sensitivity or allergy to any ingredient in the supplement or placebo. Each participant was questioned about their dietary supplement use within the last six months. Any participant beginning a new supplement routine within the last month was asked to discontinue use and allow for a two-week washout before participation. In all other cases, participants were asked to maintain their typical supplement routine throughout the study. All subjects provided written informed consent.

Potential learning effects were addressed through a dedicated familiarization session prior to experimental testing and by use of a randomized, counterbalanced crossover design. Nine of the 17 participants began the study with KET, and 8 of the 17 began with CHO. This suggests adequate balance in condition order across visits.

#### 2.1.1. Familiarization procedure

During familiarization, participants completed questionnaires regarding caffeine intake, physical activity, and health to verify inclusion. They were briefed on the study protocols, provided written informed consent, and were familiarized with cognitive testing procedures (word encoding and recall, Dynavision Go/No-Go, and KINARM assessments). A graded exercise test was used to determine maximal oxygen uptake (VO_2max_) and velocity at VO_2max_ (vVO_2max_). A VO_2max_ of at least 55 ml/kg/minutes was required in order to mitigate potential confounding by variable levels of aerobic fitness and subsequent thermal tolerance [23]. vVO_2max_ was defined as the minimal speed at which the subject was running when VO_2max_ occurred (within 2 ml/kg/minutes of VO_2max_), so long as this speed was sustained for at least 1 minute [24]. If a subject achieved VO_2max_ during a stage that was not sustained for 1 minute, the speed of the previous stage was recorded as vVO_2max_ [25]. To standardize visit conditions, participants were asked to refrain from eating for two hours prior, and from caffeine or alcohol ingestion and exercise for 24 hours prior. Adherence to these restrictions was confirmed verbally for each visit. While the exact composition and timing (outside of the two-hour window) of the last meal before each experimental visit was not standardized, participants were instructed to replicate their dietary intake before each session and compliance was verbally confirmed. This approach was used to balance experimental control with participant feasibility while minimizing acute postprandial influences.

#### 2.1.2. Experimental testing procedure

**Figure 1.**
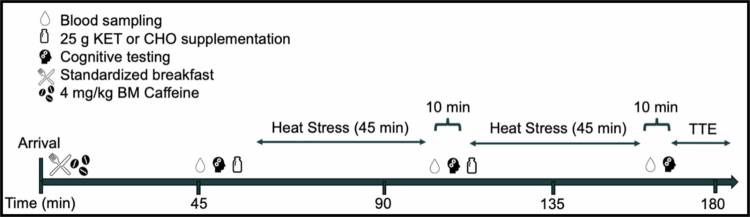
Visual outline of experimental procedure.

Figure 1 provides a visual outline of the experimental visit procedure. Upon arrival for each experimental visit, euhydration was determined via urine specific gravity (USG < 1.025) by an optical refractometer (MASTER-ALPHA, Atago, Bellevue, WA, USA). Participants then rested for 45 minutes during which time they were provided a standardized breakfast (bagel with 1 oz cream cheese and banana) and 4 mg/kg body mass of caffeine to be consumed within the first 10 minutes of the rest period.

At the conclusion of the 45-minute rest period, participants completed baseline cognitive testing and ingested their first 25 g dose of either KET ((R)-3-hydroxybutyl-(R)-3-hydroxybutyrate, Rhenium, Palm Beach, FL, USA) or CHO (Cluster Dextrin™, Glico Nutrition Co., Ltd., Osaka, Japan), which was combined with sucrose octaacetate to facilitate blinding. Thereafter, participants performed two 45-minute bouts of exercise in a heat chamber (temperature = 34 °C, and relative humidity = 45%) at 50% vVO_2max_ (average speed = 7.2 ± 0.6 kph) on a treadmill set at a 5% grade while wearing a vest weighing 20% of their BM (16.1 ± 0.8 kg). The temperature, humidity, treadmill speed, and weight vest load were selected based on prior research in a similar population [26]. After the first 45 minutes, participants completed a mid-exercise cognitive assessment outside of the heat chamber and ingested their second 25 g dose of either KET or CHO. Participants then completed the remaining 45 minutes of exercise before completing another post-exercise cognitive assessment. Blood beta-hydroxybutyrate (BHB) and glucose concentrations were measured before each series of cognitive tasks. Immediately after completing the last series of cognitive tests, participants removed their vests and performed a time-to-exhaustion test (TTE) at 90% of vVO_2max_ which concluded data collection for that experimental visit.

Throughout the treadmill protocol, participants ingested water at a fixed rate of 0.63 ml/kg BM every 15 minutes to prevent the influence of variable rates of fluid intake. Visits were separated by 7 to 14 days to prevent confounding due to acclimation to exercise-heat stress. A typical experimental visit lasted approximately three hours.

#### 2.1.3. Cognitive test procedures

For each experimental session, before exercise (timepoint 1), during their mid-exercise break (timepoint 2), and immediately post-exercise (timepoint 3), participants completed a series of cognitive tasks. During each cognitive assessment, participants completed a word encoding and recall task (Hopkins Verbal Learning Task [HVLT]), the Dynavision Go/NoGo task (D2, Dynavision International LLC, West Chester, OH, USA) [27], and a series of tasks using the KINARM End-Point system (BKIN Technologies, Kingston, Canada) [28]. With the exception of the word encoding and recall, all cognitive tasks were performed outside of the heat chamber while wearing a space blanket to minimize heat loss. In total, the cognitive assessment battery took approximately 10 minutes to complete.

### 2.2. Hopkins verbal learning task (HVLT)

During the baseline period (timepoint 1), the task administrator read aloud a series of 12 words. Once all 12 words were given, participants were instructed to verbally repeat as many words as they could remember (encoding). The average baseline coefficient of variation (CV) was 21%. During their exercise break (following cognitive tasks), participants were instructed to verbally recall as many words as possible (recall #1). Participants repeated the same procedure following their post-exercise cognitive tasks (recall #2). For each experimental session, participants were provided with a different set of words to encode and recall. Outcome measures included the percentage of correctly recalled words.

### 2.3. Dynavision

Participants completed a 60-s reaction time test on a Dynavision D2™ Reaction Board. The Dynavision D2™ is an interactive light board with 64 three-dimensional targets. For the Dynavision Go/No-Go task, participants were instructed to hit the red light as the “go” stimulus targets as fast as possible and refrain from responding to green “no-go” stimuli. Outcome measures included mean go-target RT and total number of successful target hits (total score). Go-target RT was recorded for each task run (CV = 8%).

### 2.4. KINARM

The KINARM End-Point Lab system was used to assess sensorimotor and cognitive function. This setup provides precise, simultaneous measurements of upper limb kinematics and eye movements in a horizontal augmented reality environment.

*Object Hit and Avoid.* The Object Hit and Avoid (OHA) task assessed rapid response execution and inhibition during complex continuous task performance [29]. Participants used virtual paddles to hit target objects (*n* = 200) while avoiding distractors (*n* = 100) as they moved toward them. Object drop rate and total number of simultaneous falling objects gradually increased making the task more difficult as time elapsed. Outcome measures included total accuracy (%), percentage of target hits, percentage of distractors hit, and d′. d′ was calculated as d′ = Z(hit rate) − Z(false alarm rate), where hit rate reflects correct identification of target stimuli and false alarm rate reflects incorrect hits of distractor objects. This metric provides an index of stimulus discrimination independent of response bias [30]. Total accuracy (CV = 5%) and target hits were recorded (CV = 5%).

*N-Back Task.* The 1-back and 2-back tasks were used to evaluate sustained attention and working memory [31]. Participants responded using a handheld two-button response box, pressing right if the current shape matched the one from one (1-back) or two (2-back) trials prior (target) or left if it was different (non-target). Each task consisted of 100 trials. For each *n*-back task condition (1-back, 2-back) primary outcome measures included: (1) total accuracy (% correct responses), (2) target accuracy (% correct responses to target stimuli), (3) non-target accuracy (% correct responses to non-target stimuli), (4) RT for both target and non-target stimuli, (5) coefficient of variation of reaction time (CVRT) for both target and non-target responses, and (6) d′. For the 1-back, the average baseline CV was 4% and 20% for accuracy and target accuracy, respectively. For the 2-back, the average baseline CV was 9% and 37% for accuracy and target accuracy, respectively. Baseline variability was also assessed for RT and CVRT measures to characterize within-subject reliability across visits.

#### 2.4.1. Taste matching

At the conclusion of all study visits, participants were asked to indicate whether they thought they received KET or CHO at each visit to assess the blinding protocol. Participants were not informed of the accuracy of their assumptions during the study.

#### 2.4.2. Time-to-exhaustion

Time-to-exhaustion (TTE) was performed at 90% vVO_2max_ following approximately 90 minutes of sustained, loaded exercise in a hot environment, a context expected to amplify physiological strain. The test was continued until volitional exhaustion, defined as the point at which the participant could no longer continue exercise at the prescribed treadmill velocity despite standardized verbal encouragement. To assess potential session-related learning or familiarization effects, TTE duration was compared between experimental session 1 and session 2 using a paired-samples t-test. Supplement effects on TTE duration were evaluated separately using a paired-samples t-test comparing KET and CHO.

#### 2.4.3. Physiological parameters

*Blood Glucose and Beta-Hydroxybutyrate (BHB).* Baseline blood glucose and BHB concentrations were evaluated postprandially before exercise (timepoint 1). Measurements were then repeated at the end of the first (timepoint 2) and second (timepoint 3) bouts of exercise at each experimental visit. Capillary blood samples were taken from the fingertip to analyze blood glucose and BHB concentrations (Keto-Mojo Meter 2; Napa, CA, USA).

*Heart Rate.* Participants were fitted with a chest-strap heart rate monitor (Polar H10, Polar Electro, Kempele, Finland). Heart rate was monitored continuously throughout the entire experimental visit. Mean (HR_mean_) and maximum (HR_max_) heart rate for each 45-minute exercise bout was recorded.

*Core Body Temperature (CBT)*. CBT was monitored continuously throughout the entire experimental visit via a rectal thermometer inserted to a minimum of six inches (Model 401, Yellow Springs Instruments, Yellow Springs, OH, USA). Mean (CBT_mean_). Maximum (CBT_max_) CBT for each 45-minute exercise bout was recorded. Exercise was terminated if a CBT of 39.9 °C was achieved or if any other physiological or psychological symptoms indicated the subject should discontinue exercise for safety.

#### 2.4.4. Statistical analysis

All statistical analyzes were conducted using MATLAB 2025b with built-in functions from the Statistics and Machine Learning Toolbox. Coefficients of variation (CVs) were calculated to characterize within-subject variability across visits for cognitive and physical performance outcomes. For each variable, CVs were derived from repeated baseline measurements and expressed as the within-subject standard deviation divided by the mean × 100. To further examine potential learning or practice effects that may have contributed to observed variability, baseline performance across visits was evaluated with mixed-effects models including visit/session as a fixed effect.

Object Hit and Avoid, Dynavision, blood metabolites, and average heart rate were analyzed using 2 × 2 linear mixed-model analysis of covariance (ANCOVA), with fixed effects for supplement condition (KET, CHO), timepoint (timepoint 2, timepoint 3), and the supplement × timepoint interaction, and participant included as a random effect. Baseline values were included as covariates to account for individual differences in presupplement performance or physiology, and session number was included as a covariate to account for potential learning or order effects across experimental visits.

*N*-back performance was analyzed using a 2 × 2 × 2 linear mixed-model ANCOVA with fixed effects for supplement condition (KET, CHO), timepoint (timepoint 2, timepoint 3), *n*-back task condition (1-back, 2-back), and their interactions. Baseline performance, session number, and session × *n*-back task condition were included as covariates to account for presupplementation intersubject variability and potential variations in practice effects from session 1 to session 2 as well as among *n*-back conditions. Participant was modeled as a random effect.

Maximal core temperature during exercise was analyzed using a 2 × 2 linear mixed-model ANCOVA with fixed effects for supplement condition, exercise bout, and their interaction, with session included as a covariate and participant as a random effect.

Time-to-exhaustion (TTE) duration and HR_max_ during the TTE test were analyzed using paired-samples t-tests to compare KET and CHO. A separate paired-samples t-test was used to examine potential session-related learning effects for TTE duration by comparing performance from session 1 and session 2, irrespective of supplement assignment. In addition, a smallest worthwhile change (SWC) analysis was performed for TTE duration, calculated as 0.25 × the standard deviation of the CHO condition. Individual difference scores (KET-CHO) exceeding the SWC were classified as meaningful improvements in performance.

Model-derived estimated marginal means (EMMs) were calculated for each condition, timepoint, and task where appropriate. These values account for fixed effects and covariates (baseline performance and session number), as well as the random effect of participant, and represent the predicted marginal means from the fitted mixed-effects models. EMMs were used for all pairwise comparisons and are presented throughout the Results. When significant main or interaction effects were detected, post hoc pairwise contrasts were performed using effect-specific EMMs (collapsed over nonsignificant levels) with Holm-adjusted *p*-values. Effect sizes for pairwise contrasts are reported as Hedges' g (*g*), calculated from model-derived estimates and pooled standard deviations. Statistical significance was accepted at *α* = 0.05.

## 3. Results

### 3.1. Cognitive performance

#### 3.1.1. Word encoding and recall

Mixed model ANCOVA analyzes controlling for baseline performance and session number failed to reveal any significant interaction, main effects of supplement condition, or main effects of time were observed for percent of words recalled during the HVLT (*p*'s > 0.05).

#### 3.1.2. Dynavision

Mixed model ANCOVA analyzes controlling for baseline performance and session number failed to reveal any significant interactions, main effects of supplement condition, or main effects of time were observed for Dynavision total score or RT (*p*'s > 0.05).

#### 3.1.3. Object hit and avoid (OHA)

Mixed model ANCOVA analyzes controlling for baseline performance and session number failed to reveal any significant interactions, main effects of supplement condition, or main effects of time for OHA accuracy, target hit (%), distractors hit (%), or d′ (*p*'s > 0.05).

#### 3.1.4. N-Back task

**Figure 2.**
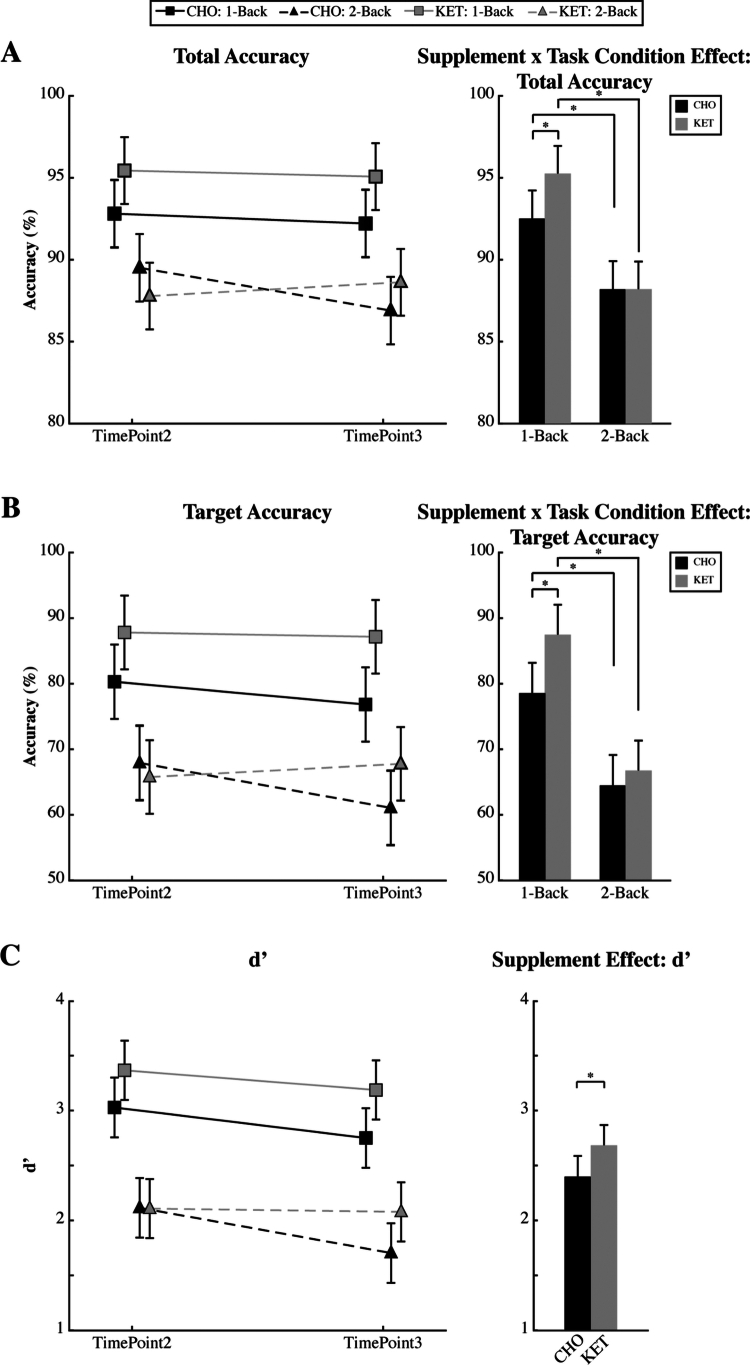
*N*-back performance across supplement condition, timepoint, and task condition (plotted estimated marginal means controlling for baseline performance and session number). Panel A: Total Accuracy. Panel B: Target Accuracy. Panel C: d prime.

Key *n*-back outcome measures across supplement condition, *n*-back task condition, and timepoints are shown in Figure 2. Mixed-effects modeling controlling for baseline performance and session number revealed a significant supplement condition × *n*-back task condition interaction effect for overall *n*-back accuracy (F [1,125] = 7.10, *p* = 0.009, pη^2^ = 0.05). Post hoc paired contrasts revealed that following CHO, participants demonstrated significantly worse total accuracy on the 2-back (EMM = 88.20%) compared to the 1-back (EMM = 92.51%) irrespective of timepoint (EMM diff = −4.31%, 95% CI: [−5.93 to −2.69], *p* < 0.001, *g* = 1.3). Similarly, following KET, participants demonstrated significantly worse total accuracy on the 2-back (EMM = 88.20%) compared to the 1-back (EMM = 95.26%) irrespective of timepoint (EMM diff = −8.86, 95% CI: [−11.67 to −6.05], *p* < 0.001, *g* = 2.6). When comparing within-task conditions, irrespective of timepoint, participants demonstrated significantly greater total accuracy on the 1-back following KET compared to CHO (EMM diff = 2.75%, 95% CI: [1.13 to 4.37], *p* = 0.001, *g* = 0.8). There was no significant difference in 2-back performance between KET and CHO (EMM diff = 0.003%, 95% CI: [−1.62 to 1.62], *p* = 0.990, *g* = 0.0).

A significant supplement condition × *n*-back task condition interaction was observed for target object accuracy (F [1,125] = 4.20, *p* = 0.040, pη^2^ = 0.03). Post-hoc paired contrasts revealed that following CHO, participants demonstrated significantly worse target object accuracy on the 2-back (EMM = 64.48%) compared to the 1-back (EMM = 78.56%) irrespective of timepoint (EMM diff = 14.08%, 95% CI: [−18.75 to −9.42], *p* < 0.001, *g* = 1.5). Similarly, following KET, participants demonstrated significantly worse target object accuracy on the 2-back (EMM = 66.75%) compared to the 1-back (EMM = 87.49%) irrespective of timepoint (EMM diff = -23.77%, 95% CI: [−31.82 to −15.72], *p* < 0.001, *g* = 2.5). When comparing within-task conditions, irrespective of timepoint, participants demonstrated greater 1-back target accuracy following KET compared to CHO (EMM diff = 8.92%, 95% CI: [4.26 to 13.59], *p* < 0.001, *g* = 0.9). However, there was no significant difference in target object accuracy on the 2-back task between KET and CHO (EMM diff = 2.29%, 95% CI: [−2.42 to 6.99], *p* = 0.340, *g* = 0.2).

A significant main effect of time was observed for target object RT (F [1,125] = 7.12, *p* = 0.01, pη^2^ = 0.04). Post hoc paired contrasts revealed that irrespective of *n*-back task condition or supplement condition, participants demonstrated faster RT to target objects at timepoint 3 (EMM = 474.2 ms) compared to timepoint 2 (EMM = 487.5  ms; EMM diff = -13.2  ms, 95% CI: [−26.1 to −0.4], *p* = 0.040, *g* = 0.2).

A significant time × *n*-back task condition effect was observed for target object CVRT (F [1,125] = 7.12, *p* = 0.01, pη^2^ = 0.05). Post hoc paired comparisons revealed that at timepoint 2, participants had no significant difference in target object CVRT on the 2-back (EMM = 19.05%) or 1-back (EMM = 17.91%) irrespective of supplement condition (EMM diff = 1.43%, 95% CI: [−3.84 to 0.83], *p* = 0.23). However, at timepoint 3 participants demonstrated significantly greater target CVRT on the 2-back (EMM = 22.20%) compared to the 1-back (EMM = 16.40%) irrespective of supplement condition (EMM diff = 5.80%, 95% CI: [3.46 to 8.13], *p* < 0.001, *g* = 1.2). When comparing within task condition, irrespective of supplement condition, participants demonstrated similar target object CVRT at timepoint 3 and timepoint 2 (EMM diff = −1.51%, 95% CI: [−3.84 to 0.83], *p* = 0.21, *g* = 0.3).

A significant supplement condition × *n*-back task condition interaction was observed for non-target RT (F [1,125] = 4.17, *p* = 0.04, pη^2^ = 0.03). Post-hoc comparisons revealed that following CHO participants demonstrated significantly greater non-target RT on the 2-back (EMM = 493.8  ms) compared to the 1-back (EMM = 423.5  ms) irrespective of time (EMM diff = 70.3 ms, 95% CI: [50.3 to 90.3], *p* < 0.001, *g* = 1.7). Similarly, following KET, participants demonstrated significantly greater non-target RT on the 2-back (EMM = 507.8 ms) compared to the 1-back (414.4 ms) irrespective of time (EMM diff = 111.7 ms, 95% CI: [77.1 to 146.3], *p* < 0.001, *g* = 2.7). Comparing within task condition, there were no significant differences in non-target RT between KET and CHO on the 1-back (EMM diff = −9.1 ms, 95% CI: [−29.2 to 11.0], *p* = 0.370, *g* = 0.2) or 2-back (EMM diff = 11.1 ms, 95% CI: [−9.0 to 21.1], *p* = 0.280, *g* = 0.3).

Additionally, a significant time × *n*-back task condition interaction was observed for non-target RT (F [1,125] = 4.13, *p* = 0.040, pη^2^ = 0.03). Post-hoc paired contrasts revealed that at timepoint 2 participants demonstrated significantly greater non-target RT on the 2-back (EMM = 497.2 ms) compared to the 1-back (EMM = 426.8  ms) irrespective of supplement condition (EMM diff = 70.4  ms, 95% CI: [50.5 to 90.4], *p* < 0.001, *g* = 2.2). Similarly, at timepoint 3 participants demonstrated significantly greater non-target RT on the 2-back (EMM = 501.4 ms) compared to the 1-back (EMM = 411.1 ms) irrespective of supplement condition (EMM diff = 90.2 ms, 95% CI: [70.3 to 110.3], *p* < 0.001, *g* = 2.2). When comparing within task condition, there were no significant difference between timepoint 3 and timepoint 2 within the 1-back (EMM diff = -15.7 ms, 95% CI: [−35.7 to 4.3], *p* = 0.120, *g* = 0.4) or the 2-back (EMM diff = 4.2 ms, 95% CI: [−15.8 to 24.1], *p* = 0.680, *g* = 0.1).

A significant main effect of supplement condition was observed for *n*-back d′ (F [1,125] = 4.33, *p* = 0.040, pη^2^ = 0.03). Post hoc paired contrasts revealed that irrespective of task condition and timepoint, participants demonstrated greater d′ (object discrimination) following KET (EMM = 2.69) compared to CHO (EMM = 2.40; EMM diff = 0.29, 95% CI: [0.15 to 0.45], *p* = 0.001, *g* = 0.6).

Mixed-effect modeling failed to reveal significant interactions, main effects of supplement condition, or main effects of time for nontarget accuracy and nontarget CVRT (*p*'s > 0.05).

### 3.2. Experimental heart rate

Mixed model ANCOVA analyzes controlling for session number revealed a significant main effect of time for average HR during the experimental exercise bouts (F [1,51] = 14.8, *p* < 0.001, pη^2^ = 0.23). Post-hoc paired contrasts revealed that compared to the first 45 minutes exercise bout (EMM = 155.4 beats·min^−1)^participants demonstrated a significantly greater average HR (EMM = 168.8 beats·min^−1^) during the second 45 minutes bout (EMM diff = 8.0 beats·min^−1^, 95% CI: [4.6 to 11.5] *p* < 0.001, *g* = 1.2) irrespective of supplement condition. No significant interaction (*p* = 0.470) or main effect of supplement condition (*p* = 0.740) were observed.

#### 3.2.1. Core body temperature

Mixed model ANCOVA analyzes controlling for session number revealed a significant main effect of time for average CBT (F [1,51] = 257.81, *p* < 0.001, pη^2^ = 0.84). Paired contrasts revealed that compared to the first 45 minutes exercise bout (EMM = 37.0 °C), participants had a significantly greater average CBT (EMM = 39.1 °C) during the second 45 minutes bout of exercise (EMM diff = 2.1 °C, 95% CI: [1.9 to 2.3], *p* < 0.001, *g* = 5.9), irrespective of supplement condition. No significant interactions or main effect of supplement condition were observed for average CBT (*p*'s > 0.05).

#### 3.2.2. Blood glucose

**Figure 3.**
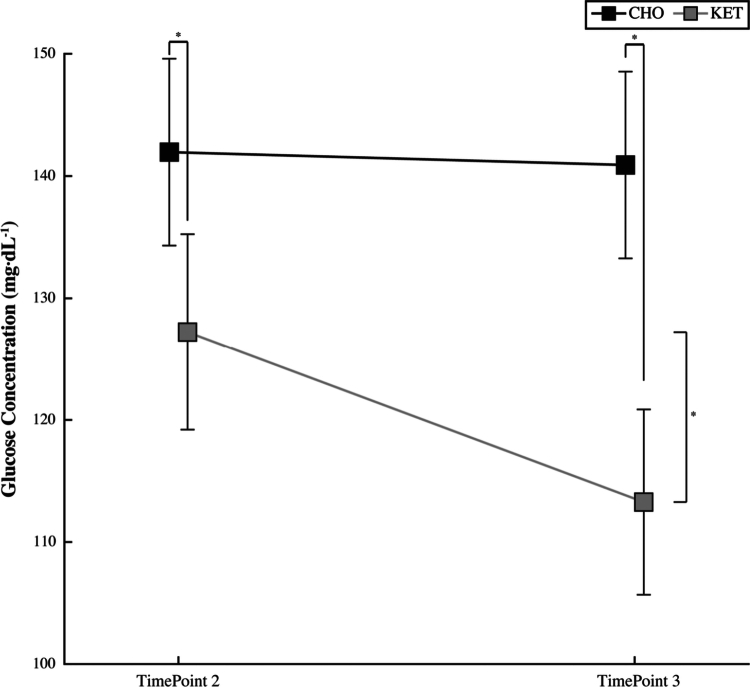
Blood glucose concentration by time point and condition (plotted estimated marginal means controlling for presupplement baseline levels and session number).

Blood glucose concentrations across supplement condition and timepoints are shown in Figure 3. Mixed-effects modeling analyzes controlling for baseline concentrations and session number revealed a significant supplement condition × time interaction for blood glucose concentrations (F [1,48] = 4.42, *p* = 0.040, pη^2^ = 0.08). Post hoc paired contrasts revealed that at timepoint 2 after taking KET participants had significantly less blood glucose (EMM = 127.2 mg·dL^−1^) compared to taking CHO (EMM = 142.0 mg·dL^−1^; EMM diff = -14.7 mg·dL^−1^, 95% CI: [−23.9 to −5.6], *p* = 0.002, *g* = 1.3). Similarly, at timepoint 3, compared to CHO (EMM = 140.9 mg·dL^−1^), participants demonstrated significantly less blood glucose after taking KET (EMM = 113.3 mg·dL^−1^; EMM diff = −27.6 mg·dL^−1^, 95% CI: [−36.4 to −18.9], *p* < 0.001, *g* = 2.4). When comparing within supplement condition, there was no significant difference between timepoint 2 and timepoint 3 (EMM diff = −1.1, 95% CI: [−9.9 to 7.8], *p* = 0.810, *g* = 0.1) following CHO. However, after taking KET, participants demonstrated significantly less blood glucose at timepoint 3 compared to timepoint 2 (EMM diff = −14.0, 95% CI: [−22.4 to −5.4], *p* = 0.002, *g* = 1.2).

### 3.3. Blood beta-hydroxybutyrate

**Figure 4.**
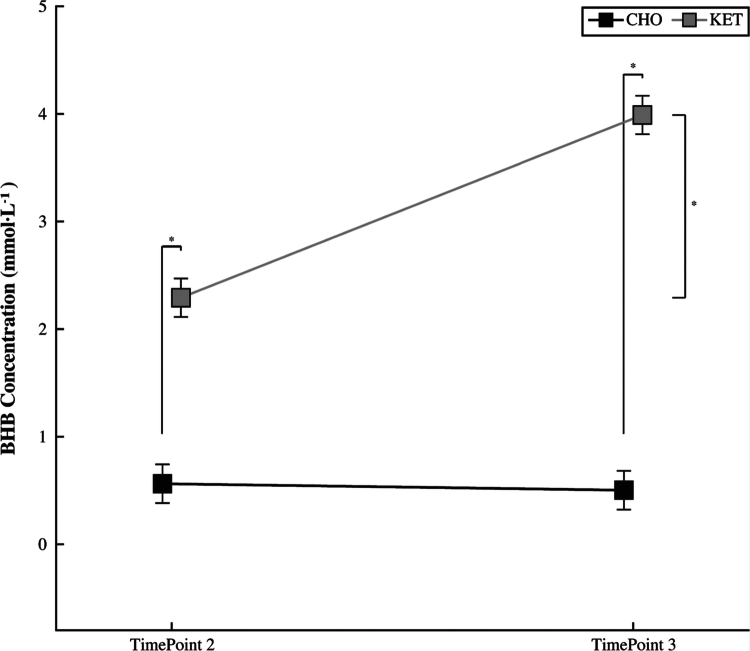
Blood beta-hydroxybutyrate (BHB) concentration by time point and condition (plotted estimated marginal means controlling for presupplement baseline levels and session number).

Blood BHB concentrations across supplement conditions and timepoints are shown in Figure 4. Mixed-effects modeling controlling for baseline concentrations and session number revealed a significant supplement condition × timepoint effect (F [1,48] = 145.94, *p* < 0.001, pη^2^ = 0.72). At timepoint 2, participants had significantly greater circulating blood BHB concentrations following KET (EMM = 2.3 mmol·L^−1^) than CHO (EMM = 0.6 mmol·L^−1^; EMM diff = 1.7 mmol·L^−1^, 95% CI: [1.5 to 1.9], *p* < 0.001, *g* = 6.0). Similarly, at timepoint 3, participants had significantly greater circulating blood BHB following KET (EMM = 4.0 mmol·L^−1^) than CHO (EMM = 0.5 mmol·L^−1^; EMM diff = 3.5 mmol·L^−1^, 95% CI: [3.3 to 3.7], *p* < 0.001, *g* = 12.0). When comparing within supplement condition, there was no significant difference between timepoint 3 and timepoint 2 following CHO (EMM diff = −0.1 mmol·L^−1^, 95% CI: [−0.3 to 0.6], *p* = 0.570, *g* = 0.2). However, participants had significantly greater circulating blood BHB at timepoint 3 compared to timepoint 2 following KET (EMM diff = 1.7 mmol·L^−1^, 95% CI: [1.5 to 1.9], *p* < 0.001, *g* = 5.9).

#### 3.3.1. Time to exhaustion test

Shapiro‒Wilk tests determined that both TTE HRmax and TTE duration satisfied assumptions of normality (*p* > 0.05), therefore were analyzed utilizing paired samples t-tests. No significant differences were observed when comparing HRmax and TTE duration between experimental session 1 and experimental session 2 (*p*'s > 0.05). Similarly, paired samples t-tests failed to reveal a significant difference in HRmax between KET and CHO (t [16] = 1.77, *p* = 0.100).

**Figure 5.**
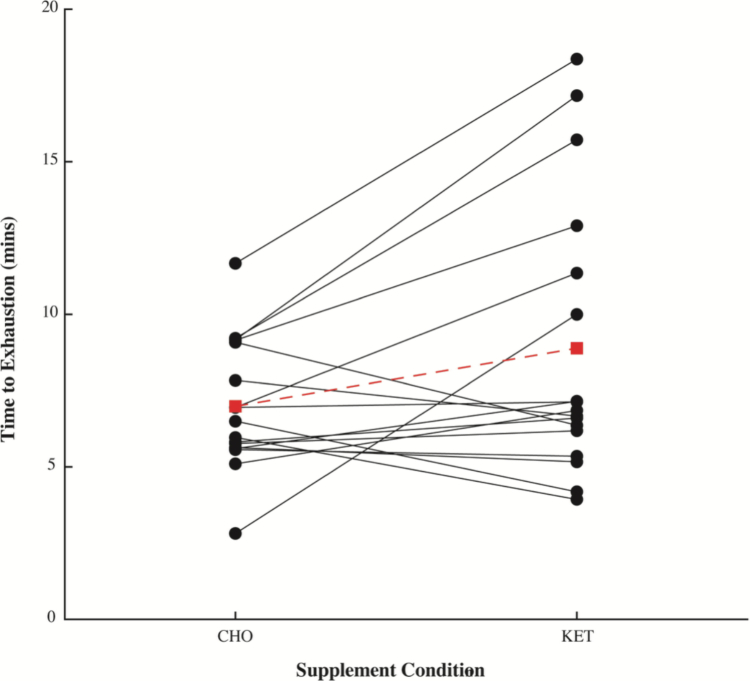
Time to exhaustion by condition. The red line indicates mean.

Individual TTE durations for each supplement condition are shown in Figure 5. Paired samples t-tests comparing TTE duration among KET (m = 8.9 ± 4.6 minutes) and CHO (m = 7.0 ± 2.1 minutes) revealed a significant difference between supplement condition (t [16] = 2.22, *p* = 0.040, *g* = 0.5). SWC calculations determined that 0.6 minutes (31.8 seconds) was a meaningful change threshold. Compared to CHO, 9/17 participants (52.9%) improved TTE duration greater than 0.58 minutes after taking KET. Conversely, 4/17 participants (23.5%) decreased TTE duration more than 0.6 minutes after taking KET, and 4/17 participants (23.5%) had no significant change in TTE duration. No significant difference in TTE duration was observed between experimental session 1 and session 2 (*p* > 0.05), suggesting no evidence of a session-related learning effect for TTE.

## 4. Discussion

The current study investigated the combined effects of KET and caffeine ingestion compared to CHO and caffeine on cognitive and physical performance during exercise-heat stress in a highly trained population [23]. Key findings indicate that ingestion of KET was associated with preserved working memory performance under high cognitive demand, as evidenced by greater 1-back total accuracy, 1-back target object accuracy, and overall greater d′ (object discrimination) compared to CHO. Additionally, KET ingestion was associated with longer TTE durations compared to CHO in the majority (but not all) of participants following prolonged loaded exercise in the heat. These findings support the hypothesis that ketone monoester supplementation may attenuate declines in cognitive performance and extend high-intensity endurance capacity during prolonged exertion in hot environments, which has particular relevance to SOF and many other tactical and/or athletic populations.

### 4.1. Cognitive performance under stress

In alignment with prior findings on the neuroprotective and energetically efficient properties of ketones [32], the present study observed that KET was associated with greater performance on select cognitive outcomes under conditions of elevated physiological stress. In the 1-back task, KET demonstrated greater total and target accuracy compared to CHO at both mid- and post-exercise time points, indicating preserved attention and stimulus discrimination during exercise-heat stress. Furthermore, KET demonstrated greater d′ within both *n*-back task conditions, indicative of better stimulus discrimination across the attention-dominant 1-back and working memory-dominant 2-back conditions. These effects suggest a consistent supplement-related difference rather than a differential temporal or task-condition response. Importantly, no within-condition changes were detected across time, indicating that the post-exercise difference reflected preserved performance in KET rather than improvement per se. These findings build upon and extend prior research suggesting that ketone metabolism can attenuate cognitive impairments under conditions such as hypoxia [17], prolonged physical exertion, [33], and diseases of metabolic syndrome [34]. Furthermore, these improvements are consistent with prior observations of increased cerebral blood flow to medial temporal, parietal, and prefrontal cortices, regions associated with working memory and sustained attention [35,36].

The present findings suggest that ketone monoester ingestion, particularly when combined with caffeine, may help preserve stimulus discrimination, sustained attention, and working memory under conditions where cognitive performance may otherwise decline. However, a key consideration when interpreting these outcomes is the potential influence of learning effects and between-visit variability. Baseline analyzes indicated a visit-related improvement in 2-back total accuracy, consistent with familiarization across repeated testing, whereas baseline 1-back accuracy and 1-back target accuracy did not demonstrate significant visit effects. Importantly, the primary supplement-related findings were observed in outcomes that were not explained by baseline visit differences, supporting the interpretation that the observed effects reflect preserved sustained attention and working memory performance rather than generalized practice effects.

These supplement effects were task-specific, as no differences were observed in word recall, Dynavision reaction time, or object hit-and-avoid performance. This reinforces the notion that the cognitive effects of KET are most apparent under higher working memory demands during central fatigue, rather than uniformly across all cognitive domains. Given the absence of time effects on lower-demand tasks, it is reasonable to conclude that either the exercise protocol or the cognitive tasks used were insufficient to impair performance in these domains or reveal supplementation-related differences. The beneficial effects observed for *n*-back d′ across task conditions highlight the relevance of ketone supplementation for preserving mission-critical functions such as stimulus discrimination in the service of sustained attention and working memory.

Although some cognitive measures demonstrated relatively high between-visit variability, the specificity of the observed effects, coupled with the absence of broad improvements across the cognitive battery, reduces the likelihood that the findings are attributable to nonspecific learning or expectancy effects alone.

### 4.2. Physiological and metabolic responses

Notably, the cognitive benefits observed in this study occurred in the absence of significant differences in physiological strain, as indicated by core body temperature and heart rate. Both conditions elicited comparable thermal responses, with average peak core temperatures approaching 39 °C, suggesting that improvements in cognitive function and TTE were not mediated by reduced thermoregulatory or cardiovascular strain. Considering that ketone ingestion resulted in markedly elevated blood BHB concentrations, increased cerebral blood flow associated with elevated BHB concentrations [13] could underlie some of the cognitive effects observed in the present study.

Given these environmental and metabolic stressors, it is also plausible that exogenous ketones supported central drive through enhanced energetic efficiency and neuroprotection, potentially preserving motivation and motor output despite hyperthermia [37]. Interestingly, the absence of a heart rate difference between conditions contrasts with some prior work reporting elevated heart rate following ketone monoester ingestion, such as found by McCarthy et al. [38]. Several factors may account for this discrepancy, including differences in participant training status, environmental conditions, blood BHB concentrations, exercise intensity and duration, and the co-ingestion strategy used in the present study. Both conditions in the current study included caffeine and were performed during prolonged loaded exercise in the heat, which likely imposed substantial sympathetic and cardiovascular strain independent of supplement condition. Under these circumstances, any ketone-related effect on heart rate may have been masked or rendered physiologically negligible relative to the effects of exercise, heat, and caffeine. This interpretation is consistent with the present data, in which HR_mean_ increased over time as expected during prolonged exercise, yet did not differ between conditions, and HR_max_ during TTE was likewise not different. Finally, in accordance with existing literature, the increases in blood BHB concentrations were associated with reductions in blood glucose concentrations [39].

### 4.3. Endurance performance

Beyond cognition, average TTE performance at 90% vVO_2max_ was 27% longer in the KET condition than in CHO. Importantly, compared to their CHO performance, 53% of participants demonstrated significantly worthwhile improvements in TTE duration (difference ≥ 0.53 minutes) after ingesting KET. Conversely, only 23.5% demonstrated worthwhile decrements in TTE duration (difference ≤ 0.53 minutes) after ingesting KET (23.5% had no meaningful change in performance). This is discordant with most of the existing literature, which has found that exogenous ketones, in the absence of more complex co-ingestion strategies, can impart ergolytic effects on high-intensity endurance performance [40]. Complex co-ingestion strategies may include simultaneous administration of ketones with carbohydrates, caffeine, or alkalizing agents such as sodium bicarbonate to mitigate potential reductions in blood pH, enhance substrate availability, or optimize energy utilization during performance [41]. The discrepancy in the literature may be due, in part, to differences in population training status, exercise modality and protocol, environmental factors, co-ingestion strategies, and nutritional background.

The TTE test in the present study was performed following 90 minutes of loaded exercise in a hot environment, a context in which both physiological and central fatigue are pronounced. While the proportion of participants demonstrating longer TTE in the KET condition is presented descriptively, this observation should be interpreted in the context of within-subject variability. Because TTE did not differ between session 1 and session 2, the longer TTE observed with KET is unlikely to be explained solely by a session-related learning or familiarization effect. Taken together, these findings suggest that ketone monoester ingestion in combination with caffeine may preserve high-intensity endurance capacity under conditions of substantial physiological strain, rather than enhance performance in rested states.

### 4.4. Limitations, strengths, and future directions

This study is not without limitations. First, while the crossover design helps control for interindividual variability, the small sample size and single-sex analysis limit generalizability. However, United States SOF units are almost exclusively comprised of males.

Second, an important limitation of the present study is the relatively high within-subject variability of several outcome measures, as reflected by elevated %CV values. This is particularly relevant when interpreting the null findings, as greater measurement variability reduces reliability and statistical sensitivity, thereby increasing the likelihood of Type II error. Accordingly, the absence of significant between-condition differences for several outcomes should not be interpreted as definitive evidence that KET had no effect, but rather that any true effect may have been small, inconsistent, or difficult to detect within the precision limits of the measures used in the present sample. This consideration is especially relevant for augmented reality test outcomes such as RT and OHA, for which no significant supplement effects were observed. Although the strength of augmented reality testing is ecological validity, it also allows for greater degrees of freedom in terms of cognitive and psycho-motor performance strategies. This may explain, in part, the notable differences between the OHA and *n*-back test where opportunities for strategic changes are limited. Future research using augmented reality tasks should be cognizant of CV's and the potential trade-off between striving for ecological validity at the cost of statistical conclusion validity. Several cognitive outcomes demonstrated substantial within-subject variability, which may have reduced statistical sensitivity and increased the likelihood of Type II error; therefore, the absence of significant effects for some outcomes should be interpreted with caution and not necessarily as evidence of no effect.

Relatedly, not all outcomes appeared to be meaningfully disrupted by the exercise-heat stress protocol itself. For some variables, including several cognitive outcomes, the lack of a clear time effect suggests that the protocol may not have imposed sufficient perturbation for KET to exert a detectable effect. In such cases, the absence of a supplement effect should be interpreted cautiously, as it may reflect either high CVs of some outcome measure, insufficient stress, or both. By contrast, outcomes that were clearly perturbed over time, such as average heart rate and core temperature, demonstrated the expected effects of prolonged exercise-heat stress, yet still did not differ between conditions, suggesting that ketone monoester plus caffeine did not meaningfully alter cardiovascular or thermal strain under the present conditions. While blood BHB concentrations were significantly elevated, the precise threshold for cognitive benefit and the interaction between circulating ketone availability and performance remain unclear.

Although our cognitive paradigm was comprehensive, evaluating multiple aspects of attention and executive control over different gradations of cognitive load, additional psychophysiological measures of cerebral hemodynamics and neuroelectric function would allow for more direct and nuanced assessment of the proposed mechanisms. In addition, the majority of the cognitive tasks took place outside the thermally stressful environment due to experimental necessity, which may have provided some temporary relief and altered the interaction between cognitive performance and hyperthermia/fatigue. To mitigate potential reductions in thermal strain during these transitions, participants were outfitted with a space blanket when traveling outside of the heat chamber. It should also be acknowledged that ketone monoester supplements are relatively costly compared to traditional supplements, which may limit widespread implementation despite some of their promising benefits. Finally, the present study did not examine the independent effects of ketone monoester, carbohydrates, and caffeine, which should be addressed in future research. It may also be worth considering a co-ingestion strategy of all three of these substances given the current study's results.

Despite these limitations, this study also presents several key strengths. First, the within-subjects, double-blind, counterbalanced design provides strong internal validity and minimizes potential confounding variables. Second, cognitive performance was assessed using a comprehensive battery of validated and operationally relevant tasks. Third, participants were endurance-trained, and the protocol included sustained exercise, load carriage, and elevated temperatures reflective of some military operations, thereby improving external validity for SOF populations. Lastly, although the current paradigm precluded the evaluation of each individual substance, caffeine is ubiquitously consumed and culturally significant within military communities, strengthening the practical applicability of the current findings [18,42]. An additional strength is that the study incorporated multiple domains of performance and physiology, allowing for a more nuanced interpretation of where ketone monoester plus caffeine may and may not exert meaningful effects. In this regard, the present results suggest a selective pattern of benefit, with improvements observed for TTE and aspects of lower-load working memory, but not across all cognitive, cardiovascular, or thermal outcomes.

Future studies should continue to examine the relationship between blood ketone concentrations and cognitive performance under diverse operational stressors (e.g. hypoxia, sleep deprivation, sustained load carriage). Future work should also prioritize outcome measures with stronger test‒retest reliability and consider larger samples to improve sensitivity for detecting smaller treatment effects. Additionally, given that most prior ketone studies report attenuation of decline rather than cognitive enhancement, the present findings raise the possibility of a context-dependent synergy between ketone monoester and caffeine during combined metabolic and thermal stress. In addition, mechanistic work using neuroimaging or cerebral hemodynamics would help clarify the relative contributions of brain perfusion versus substrate availability. More broadly, these findings should be viewed as preliminary and hypothesis-generating, helping to refine both methodological considerations and outcome selection for future studies evaluating ketone-based interventions under operationally relevant stress.

## 5. Conclusion

This study provides novel evidence that ketone monoester ingestion, when combined with caffeine, preserves working memory performance and extends high-intensity endurance capacity during exercise-heat stress in a SOF-relevant population. Differences were observed in tasks requiring greater cognitive demand and in endurance performance following prolonged loaded exercise in the heat when compared with carbohydrate and caffeine ingestion. These preliminary findings support ketone monoester supplementation as a potential countermeasure for maintaining cognitive and physical performance during exercise in the heat, and warrant further investigation in larger, field-based military studies.
